# A Snapshot of SARS-CoV-2 Genome Availability up to April 2020 and its Implications: Data Analysis

**DOI:** 10.2196/19170

**Published:** 2020-06-01

**Authors:** Carla Mavian, Simone Marini, Mattia Prosperi, Marco Salemi

**Affiliations:** 1 Emerging Pathogens Institute University of Florida Gainesville, FL United States; 2 Department of Pathology University of Florida Gainesville, FL United States; 3 Department of Epidemiology University of Florida Gainesville, FL United States

**Keywords:** covid-19, sars-cov-2, phylogenetics, genome, evolution, genetics, pandemic, infectious disease, virus, sequence, transmission, tracing, tracking

## Abstract

**Background:**

The severe acute respiratory syndrome coronavirus 2 (SARS-CoV-2) pandemic has been growing exponentially, affecting over 4 million people and causing enormous distress to economies and societies worldwide. A plethora of analyses based on viral sequences has already been published both in scientific journals and through non–peer-reviewed channels to investigate the genetic heterogeneity and spatiotemporal dissemination of SARS-CoV-2. However, a systematic investigation of phylogenetic information and sampling bias in the available data is lacking. Although the number of available genome sequences of SARS-CoV-2 is growing daily and the sequences show increasing phylogenetic information, country-specific data still present severe limitations and should be interpreted with caution.

**Objective:**

The objective of this study was to determine the quality of the currently available SARS-CoV-2 full genome data in terms of sampling bias as well as phylogenetic and temporal signals to inform and guide the scientific community.

**Methods:**

We used maximum likelihood–based methods to assess the presence of sufficient information for robust phylogenetic and phylogeographic studies in several SARS-CoV-2 sequence alignments assembled from GISAID (Global Initiative on Sharing All Influenza Data) data released between March and April 2020.

**Results:**

Although the number of high-quality full genomes is growing daily, and sequence data released in April 2020 contain sufficient phylogenetic information to allow reliable inference of phylogenetic relationships, country-specific SARS-CoV-2 data sets still present severe limitations.

**Conclusions:**

At the present time, studies assessing within-country spread or transmission clusters should be considered preliminary or hypothesis-generating at best. Hence, current reports should be interpreted with caution, and concerted efforts should continue to increase the number and quality of sequences required for robust tracing of the epidemic.

## Introduction

In December 2019, a novel coronavirus, severe acute respiratory syndrome coronavirus 2 (SARS-CoV-2), was identified in Wuhan, China, as the etiologic agent of coronavirus disease (COVID-19); as of May 2020, this virus had spread to more than 187 countries [1,2]. Common symptoms of infection include fever, cough, and shortness of breath, while severe cases are characterized by advanced respiratory distress and pneumonia, often resulting in death [3]. It is still unknown how many infected people who present mild or no symptoms can spread the virus; however, a recent study showed that in Wuhan, roughly 60% of all infections were spread by asymptomatic people [4]. This characteristic significantly thwarts the work of public health officials who are attempting to detect transmission clusters, such as the ones identified in China [5,6] and Singapore [7], through epidemiological contact tracing.

Soon after the first epidemiological and genetic sequence data of SARS-CoV-2 were made available, a glut of phylogeny-based analyses began to circulate, in scientific papers as well as on social media, discussing the origin and variants of the virus as well as the countries that may have fueled its spread [8-10]. The implications of misunderstanding the real dynamics of the COVID-19 pandemic are extremely dangerous. Ethnic or social discrimination resulting from unsupported assumptions on viral contagion—which are often amplified by irresponsible, uncontrollable communications—can be highly damaging for people and countries. Although social media platforms are often vehicles for “fake news” and hype, tremendous efforts are being made by the scientific community to provide free, up-to-date information on ongoing studies as well as critical evaluations. In particular, the US-based NextStrain [11] team has been posting real-time updates on the tracing of the epidemic by molecular analyses. Several discussions and evidence-based debates on controversial hypotheses on the epidemic have ensued (eg, the number of untraced infections in the US, the putative introduction of the virus to Italy through Germany [12], and the alleged lineage diversification in China [13], which was later criticized [14]). Another example is a recent study that identified three geographically separated variants of SARS-CoV-2 based on a phylogenetic network inferred from 160 full genomes available on March 3, 2020 [10]. This work was widely covered by the news media [15]; however, it was also highly criticized by experts in the field for its inaccurate use of phylogenetic methods, incorrect rooting of the phylogeny, and significant sampling bias [16-18]. An editorial published in *Science* [19] also highlighted how unsupported or misleading claims circulating in forums, social media, and even peer-reviewed articles have resulted from substantial overinterpretation of the available data. Hence, there is an urgent need to reframe the current debate in more rigorous scientific terms and quantitatively evaluate whether sufficient information for reliable phylogenetic and phylogeographic studies currently exists or whether gaps need to be addressed. Here, we present an in-depth longitudinal analysis of the phylogenetic information on SARS-CoV-2 genomes that became available between March and April 2020 to assess their reliability for molecular epidemiology studies.

## Methods

### Data

The GISAID (Global Initiative on Sharing All Influenza Data) database [20] was accessed on March 18, March 25, March 30, and April 24, 2020 (Table S1 in Multimedia Appendix 1 and Table S2 in Multimedia Appendix 2). Our main analyses compared the March 30 data set with the April 24 data set. After quality control of sequences that were not full genomes or contained extensive stretches of unknown nucleotides, separate sequence alignments were generated using MAFFT alignment software [21]. Each sequence alignment included all sequences collected on a given date: March 18, 794 genome sequences from 35 countries; March 25, 1662 genome sequences from 42 countries; March 30, 2608 genome sequences from 55 countries; and April 24, 8992 genome sequences from 63 countries.

### Phylogenetic Signal and Maximum Likelihood Phylogeny Inference

Before carrying out any phylogeny-based analysis of virus evolution and spatiotemporal spread, it is crucial to test the quality of the sequence data, since uneven sampling, the presence of phylogenetic noise, and the absence of a temporal signal can affect the reliability of the results (eg, ancestral state reconstructions, molecular clock calibrations) [22]. SARS-CoV-2 full genome alignments generated from sequences in GISAID [23] at different time points were analyzed as follows. Transition/transversions vs genetic distance plots were generated using DAMBE6 [24]. The presence of phylogenetic signals satisfying resolved phylogenetic relationships among sequences was evaluated by likelihood mapping analysis [25] using IQ-TREE and allowing the software to search for all possible quartets using the best-fitting nucleotide substitution model [25]. Likelihood mapping analysis estimates the likelihood of each of possible tree topology for any group of four sequences (quartet), randomly chosen from an alignment, and reports them inside an equilateral triangle (the likelihood map) where the corners represent distinct tree topologies and the center represents star-like trees. Quartets are considered to be resolved when the three likelihoods are significantly different (ie, a phylogenetic signal and most dots equally distributed in the corners indicate that the data are suitable for robust phylogeny inference). Quartets are considered to be unresolved or partially resolved when two or all three of the likelihood values are not significantly different (ie, phylogenetic noise and most dots distributed in the side or center areas indicate that the data may not be sufficient for robust phylogeny inference). Extensive simulation studies have shown that for sequences to be considered robust in terms of the phylogenetic signal, the side/center areas of the likelihood mapping must include <40% of the unresolved quartets [26]. Maximum likelihood tree reconstruction was performed in IQ-TREE based on the best-fit model chosen according to the Bayesian information criterion [27,28]. Exploration of the temporal structure (ie, the presence of a molecular clock in the data) was assessed by regression of divergence (root-to-tip genetic distance) vs sampling time using TempEst [29]. In this case, the absence of a linear trend indicates that the data do not contain a temporal signal and that the data are not appropriate for phylogenetic inference using molecular clock models. The recently developed TransPhylo software package was employed to estimate how many intermediates in the putative transmission chain connected each pair of viral sequences from two infected individuals using a transmission matrix [30]. The TransPhylo R package was used to infer the transmission matrices of SARS-CoV-2 [30].

## Results

### Sampling and Phylogeographic Uncertainty

**Figure 1:**
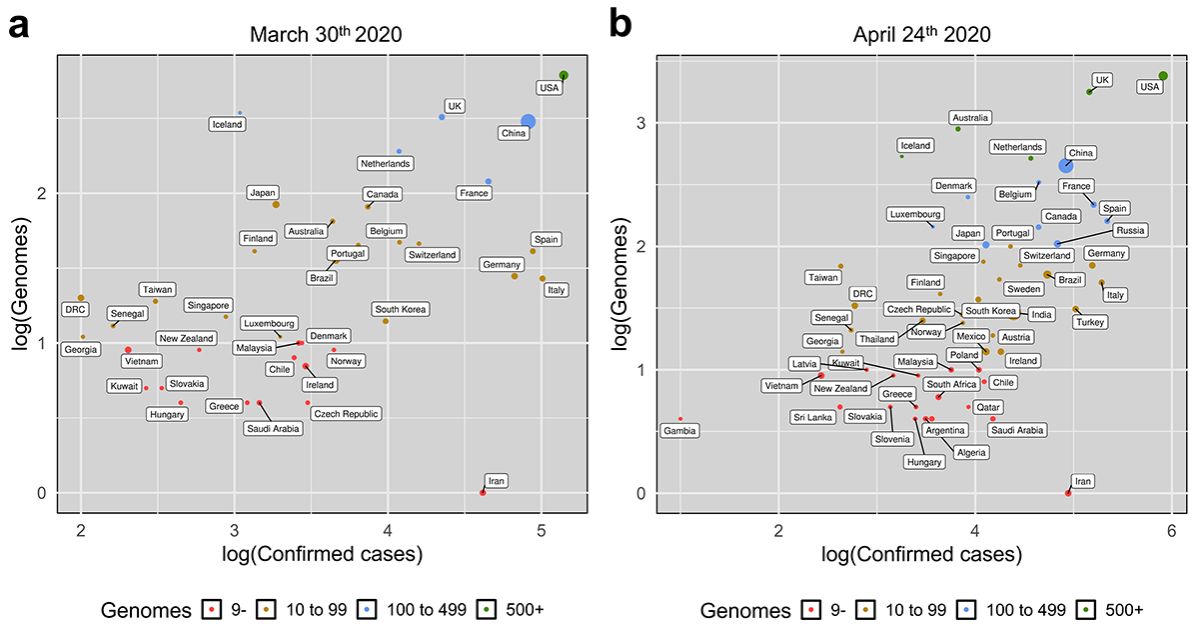
Snapshots of genomes and confirmed cases on March 30, 2020 (panel a) and April 24, 2020 (panel b). On a logarithmic scale, the x-axis reports the confirmed cases, while the y-axis reports the number of genomes +1. Each dot represents a country; the dot color indicates the number of genomes, and the dot size is proportional to the country population.

As of March 30, 2020, we compared the number of full genomes sampled per country with the number of confirmed cases at the time of sampling, as well as with the country’s total population (Figure 1). We obtained 2608 full genomes from 55 countries. During the pandemic, the number of full genomes with high coverage has been steeply increasing. By considering countries with at least 25,000 confirmed cases or 3 or more genomes in our set, we found the Spearman (rank) correlations between confirmed cases (a proxy for sampling homogeneity) and genomes per country to be fairly weak: 0.47 on March 30 and 0.52 on April 24. However, correlation could only be investigated with confirmed cases, since not all affected countries have publicly reported the total number of coronavirus tests performed. As of March 30, within the same country, sequenced genomes were usually sampled from a few hotspots; thus, these data are not necessarily representative of the whole epidemic in that country. SARS-CoV-2 full genome sequences available from patients in the United States, the country with the highest number of confirmed cases, were mainly sampled in Washington State (66%) during the early epidemic, while less than one-third (32%) available from the epicenter of the US epidemic, the state of New York. Italy, the country with the second highest number of confirmed cases, uploaded 26 genomes, 1 of which came from the Marche region, 4 from Friuli Venezia Giulia, 7 from Abruzzo, 9 from Lazio, and only 5 from Lombardy, which is the epicenter of the Italian epidemic [31] (Table S1). As of March 30, 2020, the top 10 contributors per number of genomes were the United States (n=612), Iceland (n=343), UK (n=321), China (n=300), the Netherlands (n=190), France (n=119), Japan (n=83), Canada (n=80), Australia (n=64), Spain (n=40), and Belgium (n=46). Notably, some countries uploaded a high number of genomes despite having a relatively low number of cases (eg, Georgia, Iceland, Senegal, and the Democractic Republic of the Congo). As of April 24, 2020, the top 10 contributors per number of genomes were the United States (n=2413), the United Kingdom (n=1779), Australia (n=891), Iceland (n=533), the Netherlands (n=514), China (n=449), Belgium (n=329), Denmark (n=250), France (n=217), and Spain (n=159; Table S2 in Multimedia Appendix 2).

### Phylogenetic Noise in Sequence Data

**Figure 2:**
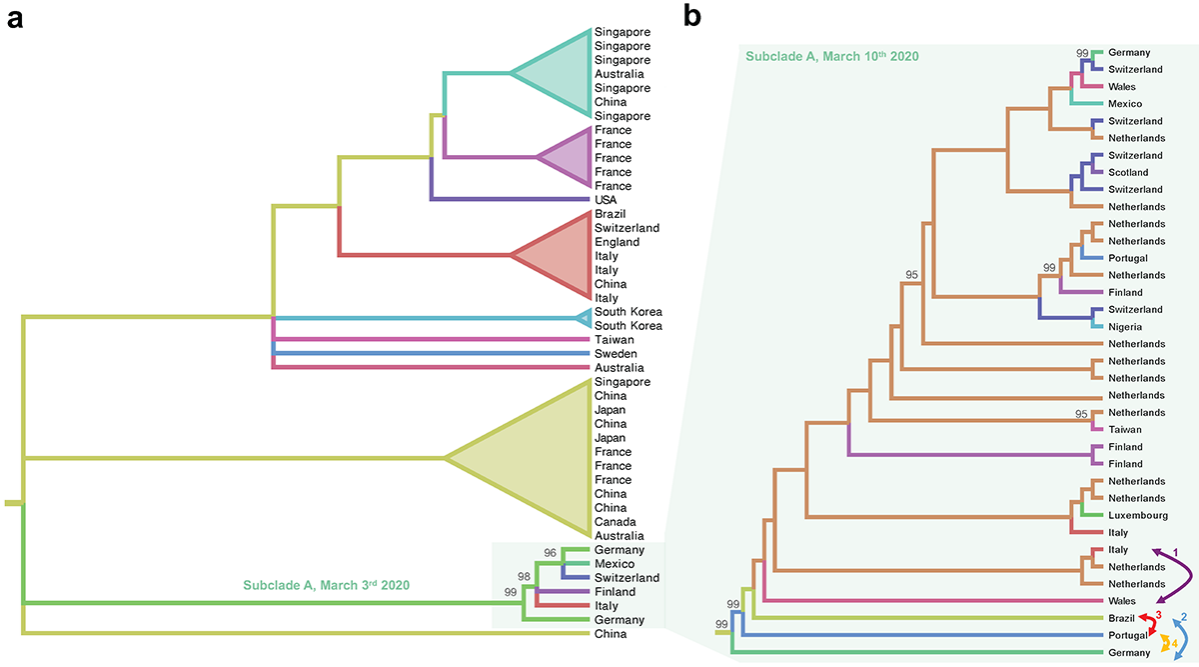
Cladograms of SARS-CoV-2 subclades. Cladograms were extracted from maximum likelihood phylogenies rooted by enforcing a molecular clock. The colored branches represent the country of origin of the sampled sequences (tip branches) and the ancestral lineages (internal branches). The numbers at the nodes indicate ultrafast bootstrap support (only >90% values are shown). (a) Cladogram of a monophyletic clade within the SARS-CoV-2 maximum likelihood tree inferred from sequences available on March 3, 2020 (Figure S1 in Multimedia Appendix 3). The subclade including sequences from Italy and Germany, named Subclade A, is highlighted. (b) Cladogram of sub-clade A of the SARS-CoV-2 maximum likelihood tree including additional sequences that became available on March 10, 2020 (Figure S2 in Multimedia Appendix 3). Each bidirectional arrow and corresponding number connects two tip branches that were switched to generate an alternative tree topology to be tested (Table 1).

Lack of resolution and uncertainty in the SARS-CoV-2 phylogenetic tree is to be expected, considering that relatively little genetic diversity can be accumulated during the first 3 months of an epidemic, even for an exponentially spreading and rapidly evolving RNA virus. Overall, the phylogenetic signal of the current data has been increasing with the number of genomes released. The percentages of unresolved quartets detected in the SARS-CoV-2 full genome alignments on March 3 and March 10 were still too high to allow reliable inferences (Figure S1 in Multimedia Appendix 3). In other words, this lack of phylogenetic signal likely resulted in overall unreliable topologies of any SARS-CoV-2 trees obtained using these data, and even clades with high bootstrap values should be interpreted with extreme caution. A preliminary maximum likelihood tree, inferred from the full genome viral sequences available on March 3, 2020, showed a well-supported cluster of European and Asian sequences (reported in Figure S2 in Multimedia Appendix 3), which contained a subclade (Subclade A, Figure 2a) including a sequence isolated in Germany that appeared to be paraphyletic (with strong bootstrap support) to an Italian sequence clustering in turn with sequences from Finland, Mexico, Germany, and Switzerland. Based on this observation (which is available on NextStrain), a heated discussion circulated on social media about a transmission event from Germany to Italy followed by further spread from Italy to other countries. However, in a new tree inferred just one week later, when more than 135 new full genome sequences were made available on GISAID [23], the direct link between Germany and Italy in Subclade A disappeared due to additional clustering of previously unsampled sequences from Portugal, Brazil, Wales, and the Netherlands (Figure 2b). In addition, the likelihood that alternative tree topologies generated arbitrarily switching branches in the tree (arrows in Figure 2b), implying different dissemination scenarios, was not significantly different (Shimodaira-Hasegawa test, Table 1) than the likelihood of the tree inferred from the real data. In other words, it is not possible with the present data to decide which branching pattern (and, therefore, which phylogeographic reconstruction) most likely represents actual dissemination routes among European countries.

As the number of available genome sequences is rapidly growing, SARS-CoV-2 full genome data sets are steadily showing less than 40% unresolved quartets in the center: 38.6% on March 18 (Figure S1c in Multimedia Appendix 3), 32.3% on March 25 (Figure S1d in Multimedia Appendix 3), 28.9% on March 30^th^ (Figure S1e in Multimedia Appendix 3), and 27.6% on April 24 (Figure S1f in Multimedia Appendix 3). This indicates that the amount of phylogenetic information can now potentially be used to define phylogenetic relationships among strains. By plotting the mean genetic distance of each sequence from the root of a phylogeny versus the sequence sampling time, we can test for a significant linear correlation, which is necessary to calibrate a reliable molecular clock [29] (Figure S3 in Multimedia Appendix 3). As expected in genomes obtained over a very short period of time (approximately 3 months) since the beginning of the outbreak, the correlation in the current data is fairly weak (Table 1). Reconstructing the phylogenetic relationships of the same European sub-clade A discussed above with the sequences available on March 18, 2020 showed a much more complex snapshot of the spread of SARS-CoV-2 (Figure S4 in Multimedia Appendix 3). A closer look at subclade A reveals that even with more genomes available, inference is biased by oversampling of some countries and undersampling of others (Figure S4 in Multimedia Appendix 3). Moreover, when estimating the number of intermediates in the putative transmission chain, we found that numerous links among samples were still missing (Figure S5 in Multimedia Appendix 3). In such a scenario, it is not advisable to extrapolate conclusions on the origin and dissemination of strains.

The phylogenetic signal is increasing in the global alignment; however, likelihood mapping per country using data from countries reporting the highest numbers of cases (United States, Italy, Spain, Germany, and France) indicates that some local data sets lacked sufficient signals up to March 30, 2020 (Figure S6 in Multimedia Appendix 3). In particular, a lack of signal was found in sequence sets from Italy (26 genomes, 45 variant sites, 0.2% of total sites in the genome, 11 parsimony informative), the United States (612 genomes, 675 variant sites, 2.3% of total sites in the genome, 158 parsimony informative) and China (300 genomes, 742 variant sites, 2.5% of total sites in the genome, 98 parsimony informative). The top 5 contributing states in the United States are Washington (405/612, 66.2%), California (45/612, 7.4%), Minnesota (33/612, 5.4%), Wisconsin (29/612, 4.7%), and Utah (22/612, 3.6%); 42 genomes (6.9%) are not labeled with a state or city. The United States data set comprised mostly sequences collected in Washington State (423/612 genomes, 69.1%). The top 5 contributing provinces in China are Shanghai (96/300, 32.0%), Guangdong (80/300, 26.7%), Hong Kong (30/300, 10.0%), Hubei (31/300, 10.3%), Hangzhou (9/300, 3.0%), and Shandong (9/300, 3.0%); 20 genomes (6.7%) are not labeled with a province or city. Neither China nor the United States showed a phylogenetic signal despite the high number of genome sequences available (Figure S6 in Multimedia Appendix 3). Contrastingly, and unexpectedly, countries with low numbers of genome sequences (Germany, Spain, and France) did show a phylogenetic signal (Figure S6 in Multimedia Appendix 3). The presence of a phylogenetic signal (<40% unresolved quartets in the center) was detected only for Germany (27 genomes, 34 variant sites, 0.2% of total sites in the genome, 15 parsimony informative), with Düsseldorf and North Rhine Westphalia being the highest contributing regions (12 and 11 genomes, respectively); Spain (40 genomes, 60 variant sites, 0.2% of total sites in the genome, 23 parsimony informative), with Madrid and Comunidad Valenciana being the highest contributing regions (18 and 10 genomes, respectively); and France (119 genomes, 155 variant sites, 0.5% of total sites in the genome, 44 parsimony informative), with Auvergne-Rhône-Alpes, Hauts de France, and Bretagne being the highest contributing regions (42, 30, and 13 genomes, respectively). Despite the presence of a phylogenetic signal in these countries, only the genomes from France also showed a temporal signal that would allow the calibration of a molecular clock and reframing of the phylogenetic and phylogeographic inferences in the spatiotemporal dimension (Figure S7 in Multimedia Appendix 3). On the other hand, the transmission matrix for France indicates that considerable links are still missing due to unsampled infected individuals, limiting the reliability of transmission cluster studies based on the sequence data (Figure S8 in Multimedia Appendix 3). When we looked almost a month later at the phylogenetic signals for the countries that reported the highest numbers of confirmed cases as of April 24, 2020, we found that these countries showed sufficient phylogenetic signals (Figure S9 in Multimedia Appendix 3). However, while France and Germany also displayed sufficient temporal signals to allow in-depth molecular epidemiology studies, at least in principle, data sets from the United States (3.9-fold increase on April 24 with respect to March 30), the United Kingdom (5.5-fold increase), and Spain (3.9-fold increase), still showed weak or no temporal signals (Figure S10 in Multimedia Appendix 3) despite the substantial increases in the number of available sequences.

**Table 1:** Testing of alternative topologies.

Alternative topology^a^	Switched branches	LogL^b^	∆L^c^	*P* value^d^
1	Italy with Wales	–45443.2	0.0000	.24
2	Germany with Brazil	–45451.5	8.3554	.16
3	Portugal with Brazil	–45443.2	0.0002	.75
4	Germany with Portugal	–45451.5	8.3197	.16

^a^Alternative topologies were obtained by switching branches in the maximum likelihood tree inferred from SARS-CoV-2 full genome sequences. 1) Italy (EPI_ISL_412973) switched with Wales (EPI_ISL_413555); 2) Germany (EPI_ISL_406862) with Brazil (EPI_ISL_412964); 3) Portugal (EPI_ISL_413648) with Brazil (EPI_ISL_412964); 4) Germany (EPI_ISL_406862) with Portugal (EPI_ISL_413648).

^b^LogL: log likelihood estimated for each alternative topology.

^c^∆L: difference between LogL and the log likelihood of the original tree.

^d^Calculated with the Shimodaira-Hasegawa test [32].

## Discussion

Characterization of transmission events is fundamental to understand the dynamics of any infectious disease. From a public health standpoint, it is crucial to be able to trace transmissions at the local level. Within-country identification of active transmission clusters would open the way to more effective public health interventions. The most optimal inference of transmission events would contain a combination of genetic and epidemiological data for a joint analysis. Indeed, transmission investigations that have been performed to date have been based on contact-tracing, epidemiological, and clinical data [33,34]. Bayesian analysis [35], which infers phylogenetic and phylogeographic patterns from a posterior distribution of trees, can facilitate comparisons of different evolutionary scenarios, aid retrieval of the correct topology, and estimate an accurate evolutionary rate using relaxed clock methods [36]. More genome sequences, sampled at different time points and from diverse geographic areas, are becoming available daily; therefore, in-depth Bayesian phylodynamic and phylogeographic analyses of the COVID-19 pandemic will soon be a viable option. However, it is important to consider the dramatic effects of inhomogeneous sampling, lack of phylogenetic signal, and missing data on phylogeographic reconstructions [37].

Published scientific data and media are currently easily accessible to a worldwide audience; proper weighing of the information being shared is more important than ever. In the first months of the epidemic, many researchers rushed to study local dynamics and to publish their findings without assessing the bias in sampling or the presence of a phylogenetic or temporal signal. As shown by our analysis, as of March 2020, the United States and Italy, the two countries with the highest numbers of confirmed cases, did not show sufficiently large or representative sampling. This finding is extremely worrisome and raises questions regarding the generalizability of the results of studies investigating the origin of the introduction of SARS-CoV-2 in Italy [8,12] or of the circulation of SARS-CoV-2 in the state of Washington in early March 2020 [9]. Rushed studies [10,13] that are acclaimed by news media despite being criticized in the literature [16-18] and on social media [14] may do more harm than good. To recapitulate the importance of examining phylogenetic information in available data before performing phylogenetic inferences that may lead to erroneous or unreliable conclusions, we propose the use of a well-established phylogenetic checkpoint pipeline (Figure S11 in Multimedia Appendix 3) [22]. The first step that researchers must take before they complete their phylogenetic studies is determining whether the data set is biased in terms of the number of genomes per given location, host, source, etc. In the specific case of SARS-CoV-2, it would be advisable to calculate the correlations between the confirmed cases and genomes per country. If this first step is completed, the second step is to build a proper codon-based alignment while ensuring that the alignment is in frame; this is extremely important when researchers study selective pressures. The third step consists of assessing the presence of a sufficient phylogenetic signal and the absence of nucleotide substitution saturation, which decreases the phylogenetic information contained in the sequences [38]. The analysis can proceed to the fourth step, determining the presence or absence of recombination, only if the previous criteria are met. Recombination can impair the phylogenetic signal [39,40] and this is another important checkpoint before inferring a phylogeny. In this study, we did not test for recombination for the SARS-CoV-2 data set, as absence of recombination in the human lineage has previously been shown [41]; however, because coronaviruses are prone to recombination events, this step should be performed as more sequences become available. Detecting the presence of a temporal signal is an additional step that must be performed before the inference of a phylogeny scaled in time. Without a correlation between genetic divergence and time, it is not possible to calibrate a molecular clock and therefore to obtain a phylogeny scaled in time, regardless of whether the method employed to date the phylogeny is Bayesian [35], maximum likelihood [42], or least-squares dating [43]. Only when all these checkpoints have been considered and given proper weight should subsequent analyses be considered by choosing adequate phylogeny inference methods.

The genomic data set available on GISAID is rapidly growing; thus, a limitation of our study is that we can only provide a snapshot of the past, and this may not reflect the most current situation. We have already shown an increase in phylogenetic and temporal signals that may allow researchers to attempt to estimate the origin and spatiotemporal dissemination of SARS-CoV-2 as long as sampling bias is properly taken into account. However, it is important to reiterate that during the month of March 2020, we deem that the molecular epidemiology data and studies were not sufficiently solid to provide a scientifically sound analysis of SARS-CoV-2 spread. Thus, we suggest that any conclusions drawn about existing lineages and the direction of viral spread that were based on the sequence data available up to March 30, 2020 should be considered preliminary and hypothesis-generating at best. The evolutionary dynamics of SARS-CoV-2 spread is revealing an unprecedented amount of information, which is essential to make policy decisions. The whole of humanity is threatened by the current pandemic, and policymakers must adjust their mitigation measures while the pandemic itself is developing. Some of the urgent answers required lie in the timely availability of abundant, high-quality genetic data not only from countries experiencing a high number of reported cases but also from countries that appear to be experiencing, at least currently, a lower number of infections.

## Appendix

Multimedia Appendix 1 Table S1. GISAID IDs of genome sequences downloaded from GISAID on March 30, 2020.

Multimedia Appendix 2 Table S2. GISAID IDs of genome sequences downloaded from GISAID on April 24, 2020.

Multimedia Appendix 3 Supplementary Figures.
