# Correction: A Snapshot of SARS-CoV-2 Genome Availability up to April 2020 and its Implications: Data Analysis

**DOI:** 10.2196/22853

**Published:** 2020-08-10

**Authors:** Carla Mavian, Simone Marini, Mattia Prosperi, Marco Salemi

**Affiliations:** 1 Emerging Pathogens Institute University of Florida Gainesville, FL United States; 2 Department of Pathology University of Florida Gainesville, FL United States; 3 Department of Epidemiology University of Florida Gainesville, FL United States

In “A Snapshot of SARS-CoV-2 Genome Availability up to April 2020 and its Implications: Data Analysis” (JMIR Public Health Surveill 2020;6(2):e19170) the authors noted errors in the supplementary material files. The previous Multimedia Appendix 3 file included outdated versions of Figure S5 and Figure S8.

The replacement version can be seen in the attached file and will appear in the online version of the paper on the JMIR Publications website on August 10, 2020, together with the publication of this correction notice. Because this was made after submission to PubMed, PubMed Central, and other full-text repositories, the corrected article has also been resubmitted to those repositories.

## Appendix

Multimedia Appendix 3 Supplementary Figures.

