# Supplementary material for: A Snapshot of SARS-CoV-2 Genome Availability up to April 2020 and its Implications: Data Analysis
Source: JMIR Public Health Surveill. 2020 Jun 1;6(2):e19170. doi: 10.2196/19170 (PMC7265655; doi:10.2196/19170)

**Supplementary Figures**

**Figure S1.** Phylogenetic signals for 169 and 331 full genomes of SARS-CoV-2. The presence of a phylogenetic signal was evaluated by likelihood mapping checking for alternative topologies (tips), unresolved quartets (center), and partly resolved quartets (edges) for the (a) 169 genome sequences available on March 3, 2020, (b) 331 available on March 10, 2020, (c) 794 available on March 18, 2020, (d) 1660 available on March 25, 2020, (e) 2608 available on March 30, 2020, and (f) 8992 available on April 24, 2020.


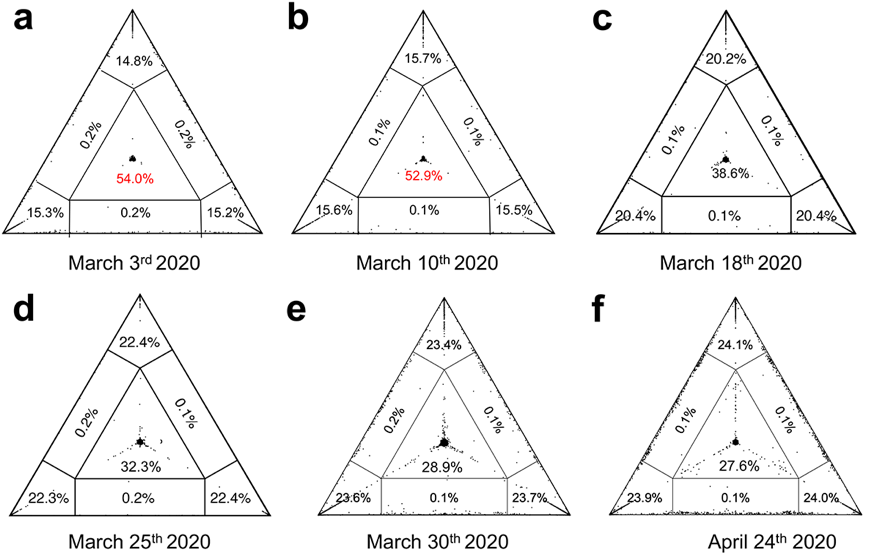


**Figure S2.** Phylogeographic cladogram of SARS-CoV-2 on March 3, 2020. Ancestral state reconstruction performed with TreeTime on 169 full genomes of SARS-CoV-2 collected on March 3, 2020. Branches are colored by country of origin.


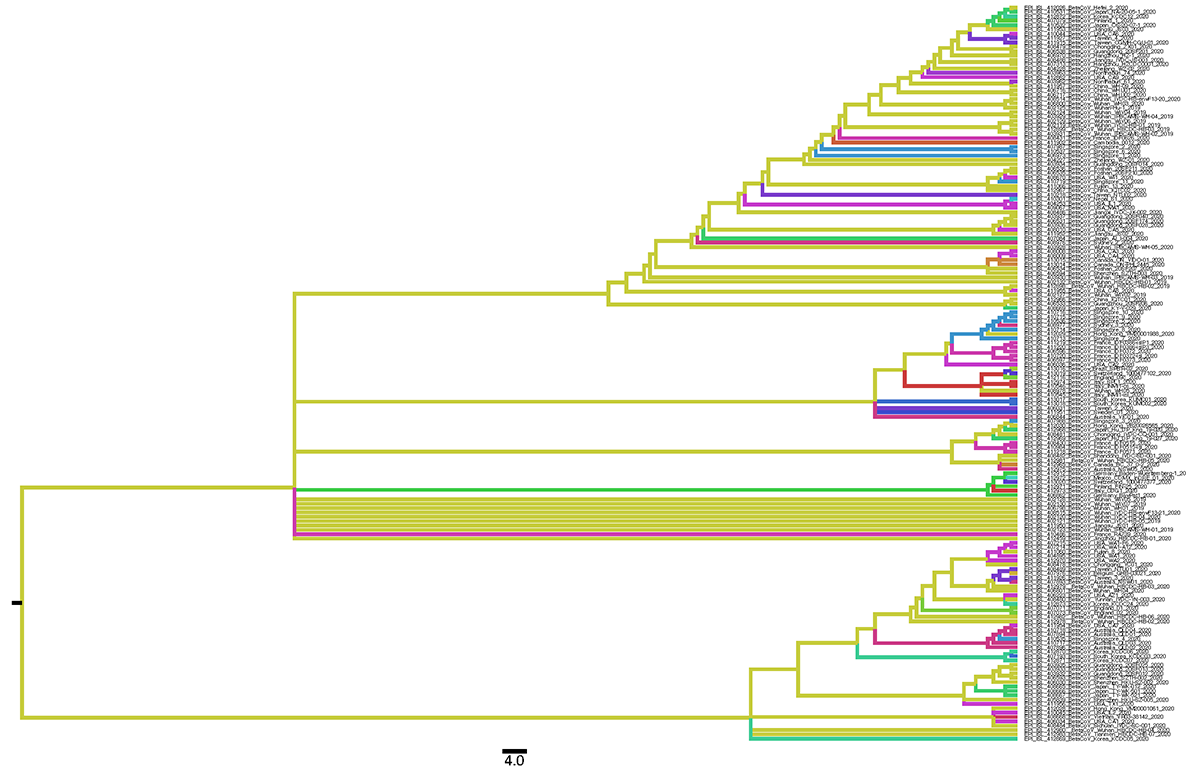


**Figure S3.** Regression analysis of temporal resolution of SARS-CoV-2 genomes on the March 18, 2020 and March 25, 2020 data sets. The plots represent the linear regression of the root-to-tip genetic distance within the maximum likelihood phylogeny against the sampling time for each taxa. Temporal resolution was assessed using the slope of the regression, with a positive slope indicating a sufficient temporal signal for data sets collected on (a) March 18, 2020 and (b) March 25, 2020. The correlation coefficient *r* is reported for each genomic fragment.


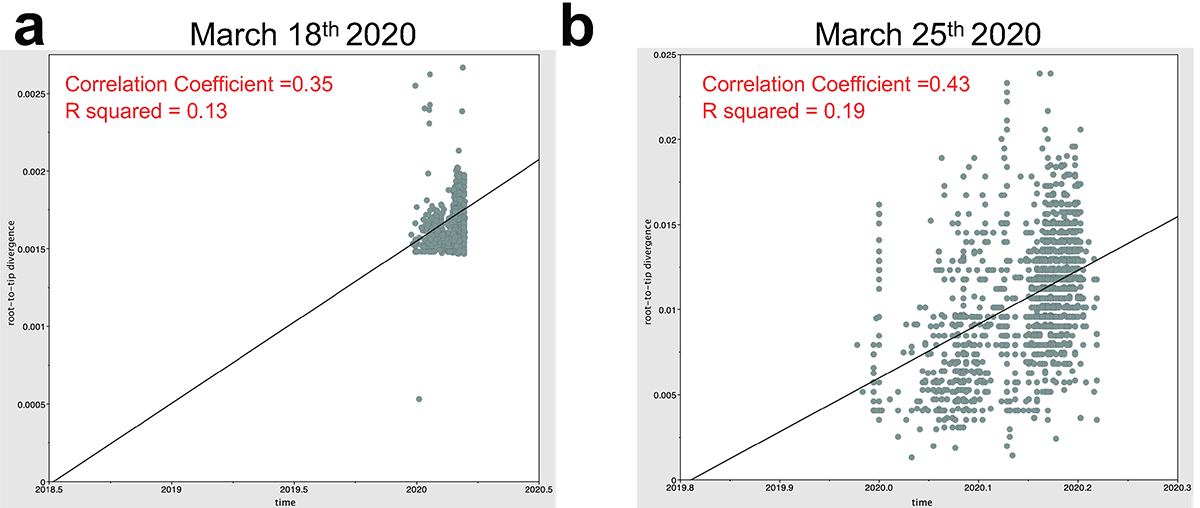


**Figure S4.** Phylogeographic reconstruction of Subclade A clade as of March 18, 2020. Ancestral state reconstruction performed with TreeTime on Subclade A with genome collected on March 18, 2020. Branches are colored by country of origin.


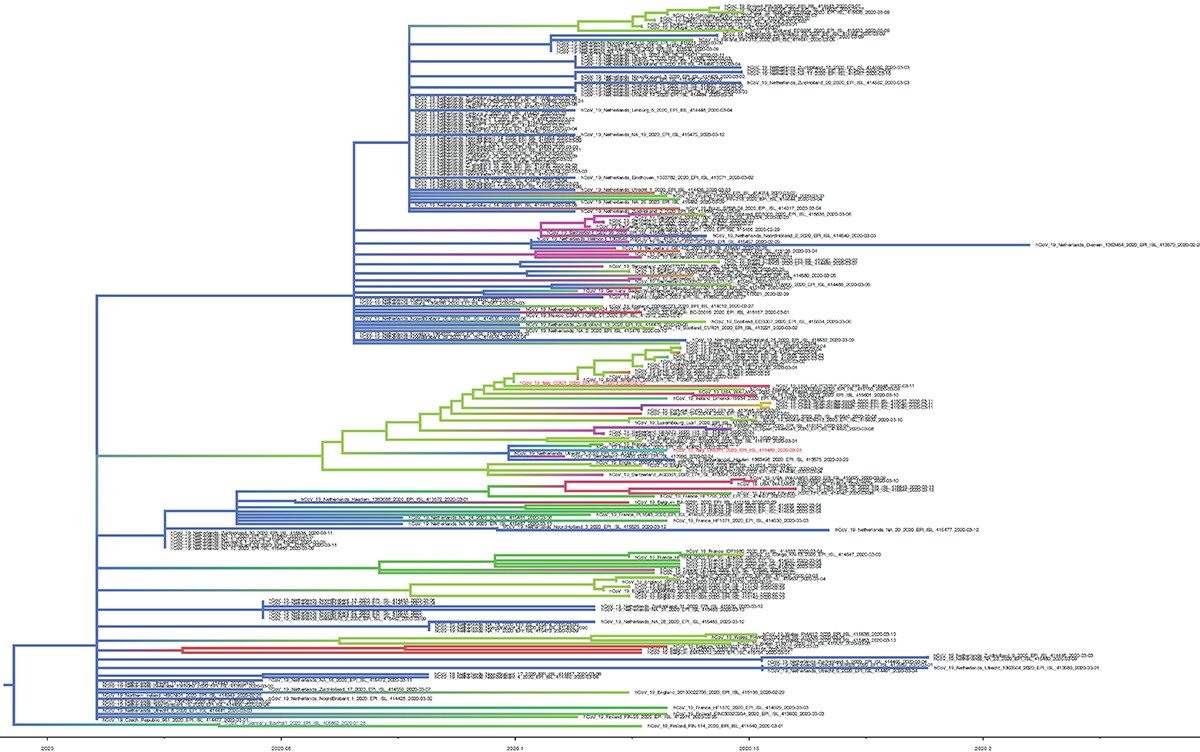


**Figure S5.** Transmission matrix of SARS-CoV-2 of Subclade A as of March 18, 2020. The transmission matrix indicating the number of intermediates for each pair of individuals in the transmission chain is given for Subclade A as of March 18, 2020. Matrix was obtained using TransPhylo with w.shape=2.5 and w.scale=2.7 as parameters (average values from preprint paper by Li et al available at www.medrxiv.org/content/10.1101/2020.02.26.20028431v1)


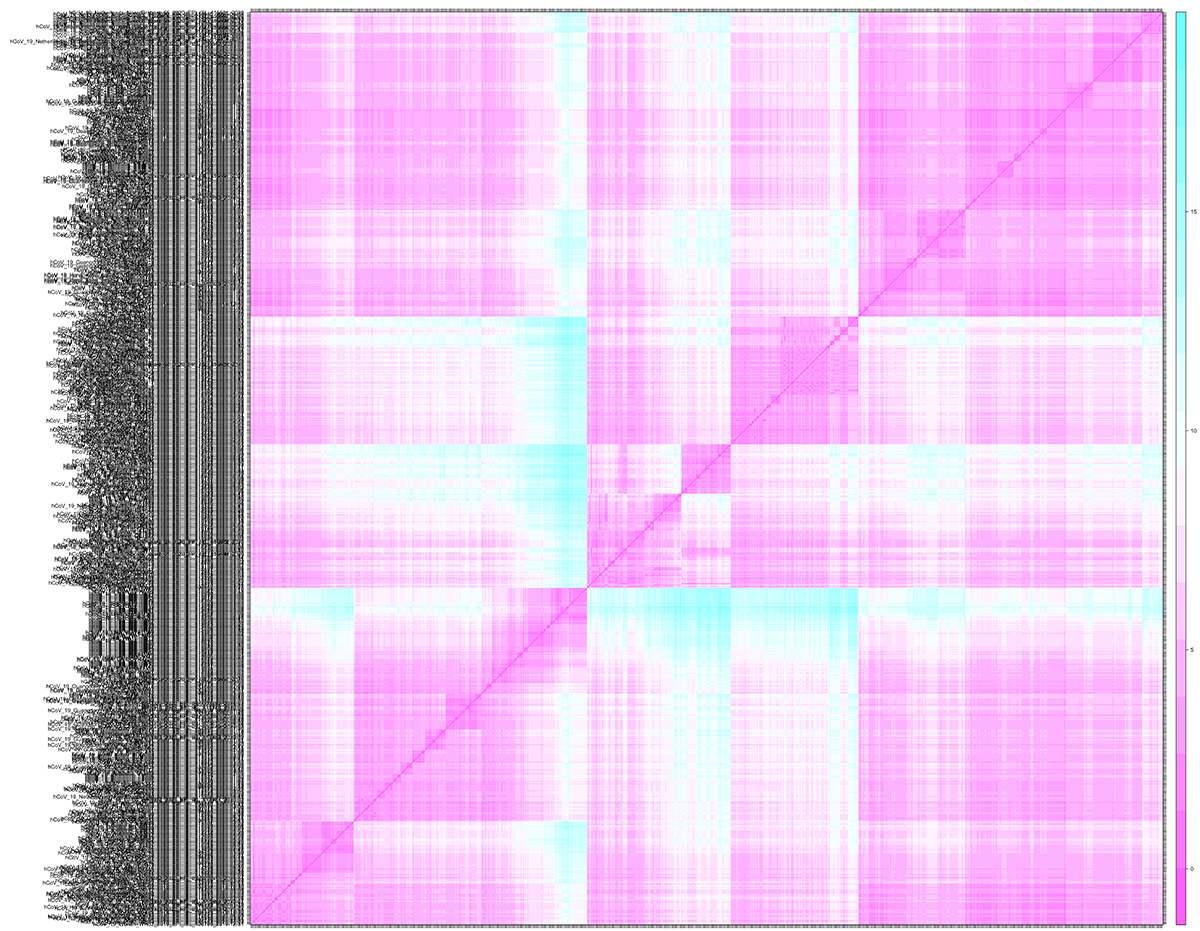


**Figure S6.** Phylogenetic signals for local SARS-CoV-2 data sets as of March 30, 2020. The presence of the phylogenetic signal was evaluated by likelihood mapping checking for alternative topologies (tips), unresolved quartets (center), and partly resolved quartets (edges) for genomes available on March 30, 2020 from (a) the United States, (b) Italy, (c) Spain, (d) China, (e) Germany, and (f) France. Presence of phylogenetic signal (<40% unresolved quartets in the center).


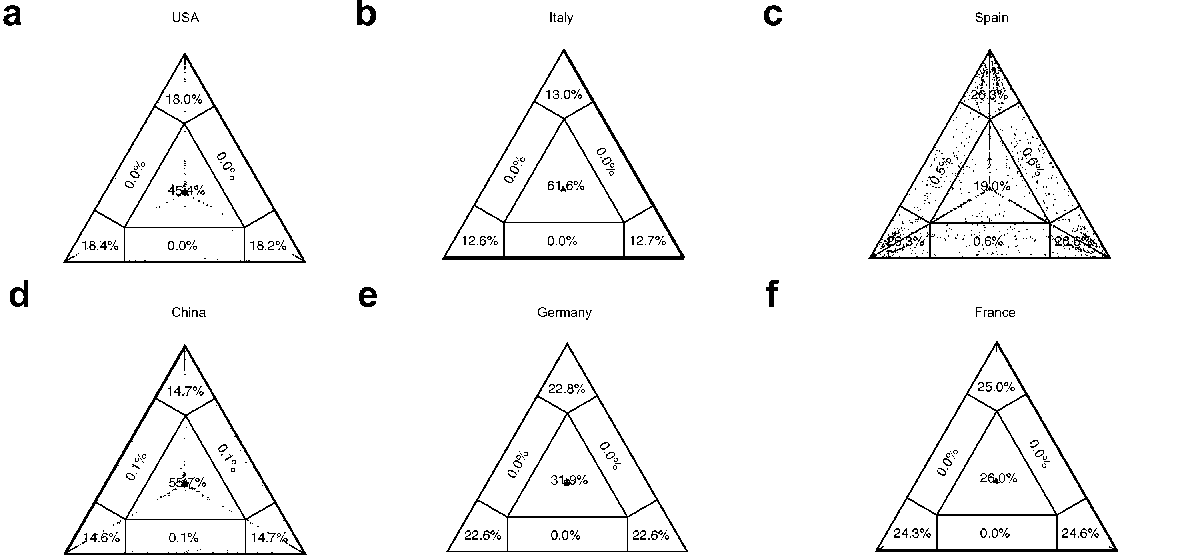


**Figure S7.** Regression analysis of temporal resolution of SARS-CoV-2 genome data sets from France, Spain, and Germany. The plots represent the linear regression of the root-to-tip genetic distance within the ML phylogeny against the sampling time for each taxa. Temporal resolution was assessed using the slope of the regression, with a positive slope indicating a sufficient temporal signal for data sets collected from (a) France, (b) Spain, and (c) Germany. The correlation coefficient *r* is reported for each genomic fragment.


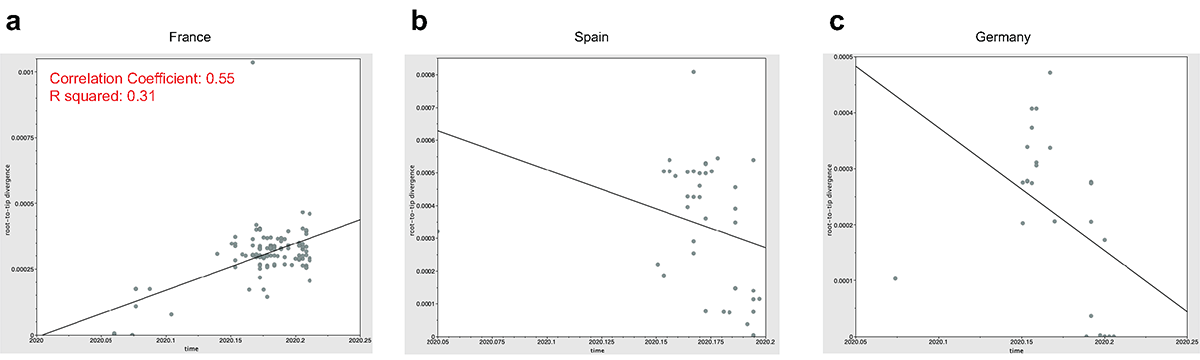


**Figure S8.** Transmission matrix of SARS-CoV-2 in France. The transmission matrix indicating the number of intermediates for each pair of individuals in the transmission chain is given for France (119 genomes as of March 30, 2020; **40,708** confirmed cases as of March 29, 2020). Matrix was obtained using TrasPhylo with w.shape=2.5 and w.scale=2.7 as parameters (average values from preprint paper by Li et al available at www.medrxiv.org/content/10.1101/2020.02.26.20028431v1)


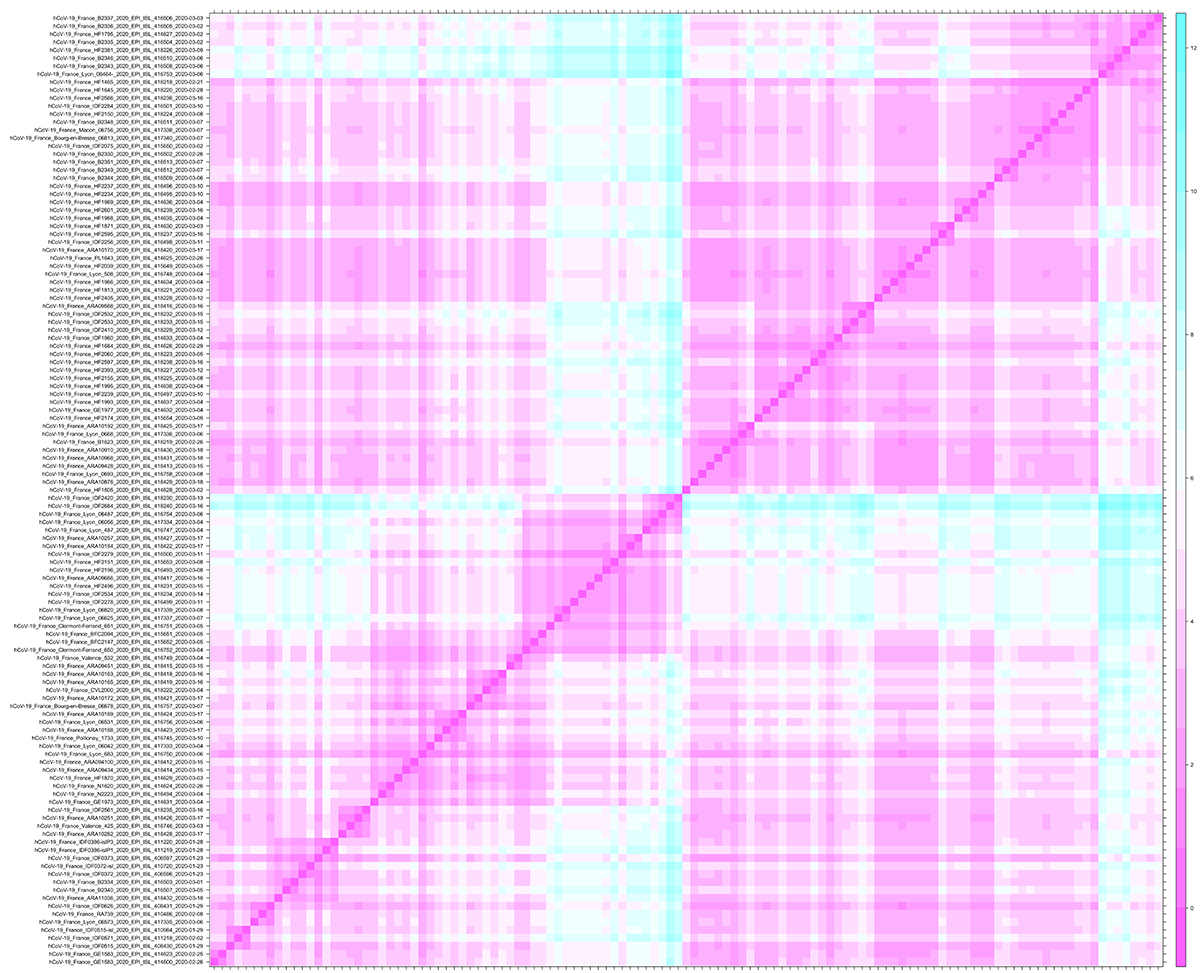


**Figure S9.** Phylogenetic signals for local SARS-CoV-2 data sets as of April 24, 2020. The presence of a phylogenetic signal was evaluated by likelihood mapping checking for alternative topologies (tips), unresolved quartets (center), and partly resolved quartets (edges) for genomes available on April 24, 2020 from (a) the United States, (b) Italy, (c) Spain, (d) China, (e) and (f) France. Presence of phylogenetic signal (<40% unresolved quartets in the center).


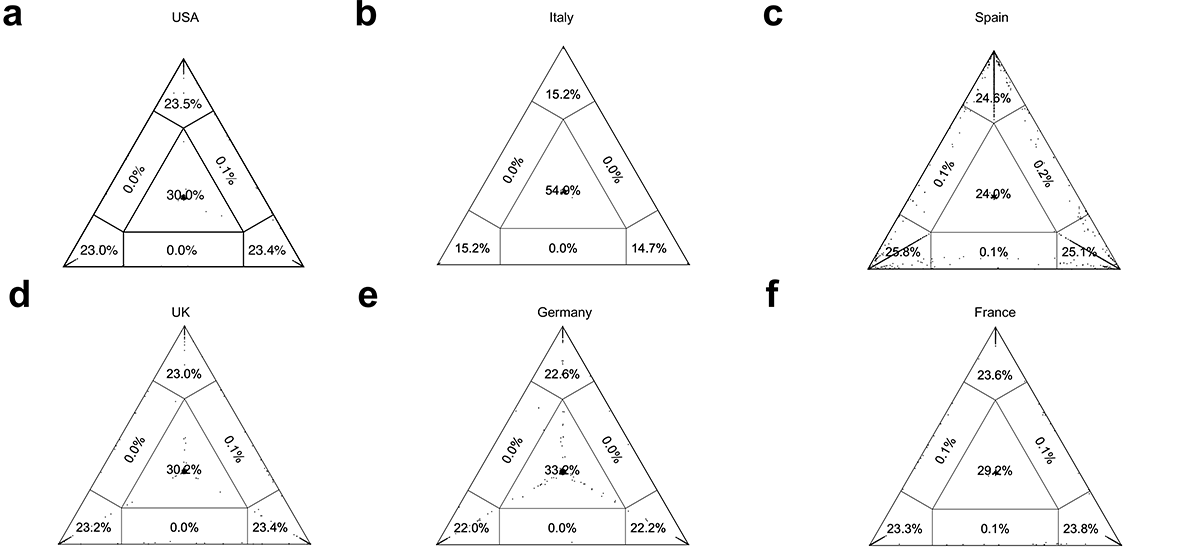


**Figure S10.** Regression analysis of temporal resolution of SARS-CoV-2 genome data sets from (a) France, (b) Germany, (c) the United States, (d) Spain, and (e) the United Kingdom. The plots represent the linear regression of the root-to-tip genetic distance within the maximum likelihood phylogeny against the sampling time for each taxa.


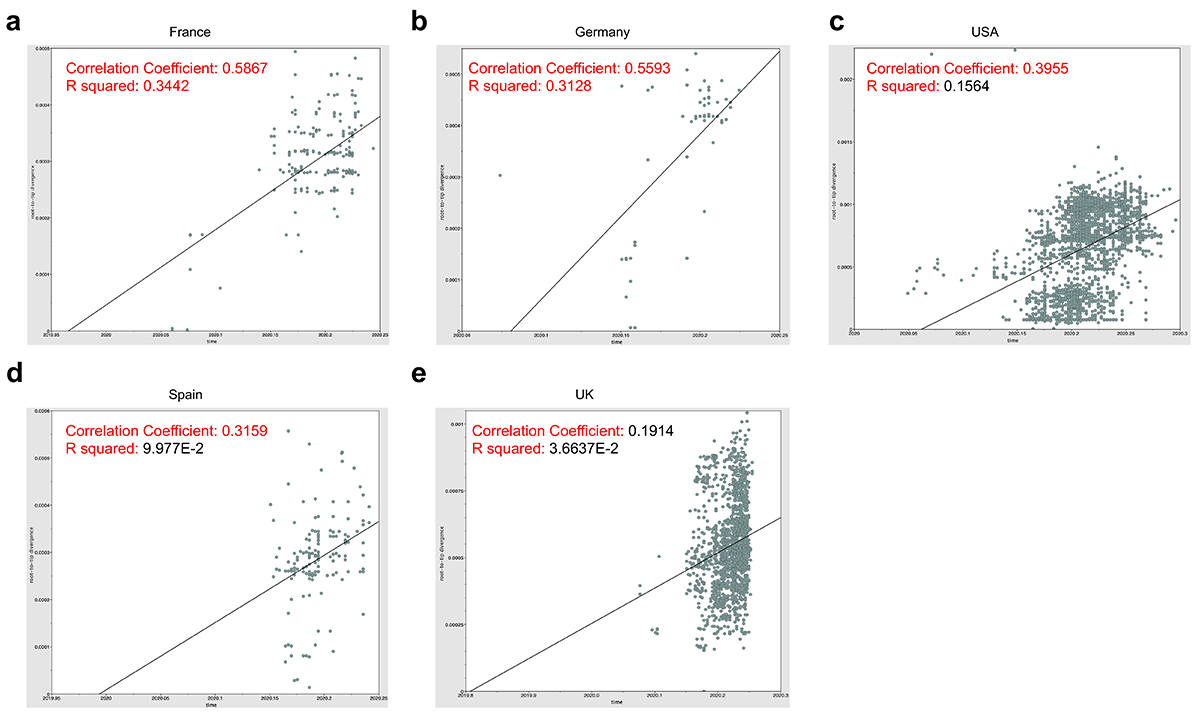


**Figure S11.** Phylogenetic checkpoint pipeline. The first step is determining whether the data set is biased in terms of the number of genomes per given location, host, source, etc. The second step is to build a proper codon-based alignment, making sure that the alignment is in frame. The third step consists of assessing the presence of a sufficient phylogenetic signal and the absence of nucleotide substitution saturation. The fourth step is determining the presence or absence of recombination. The fifth step to be performed before the inference of a phylogeny scaled in time is detection of the presence of a temporal signal. Only when all these checkpoints have been considered and given proper weight should subsequent analyses (step 6) be considered by choosing adequate phylogeny inference methods.


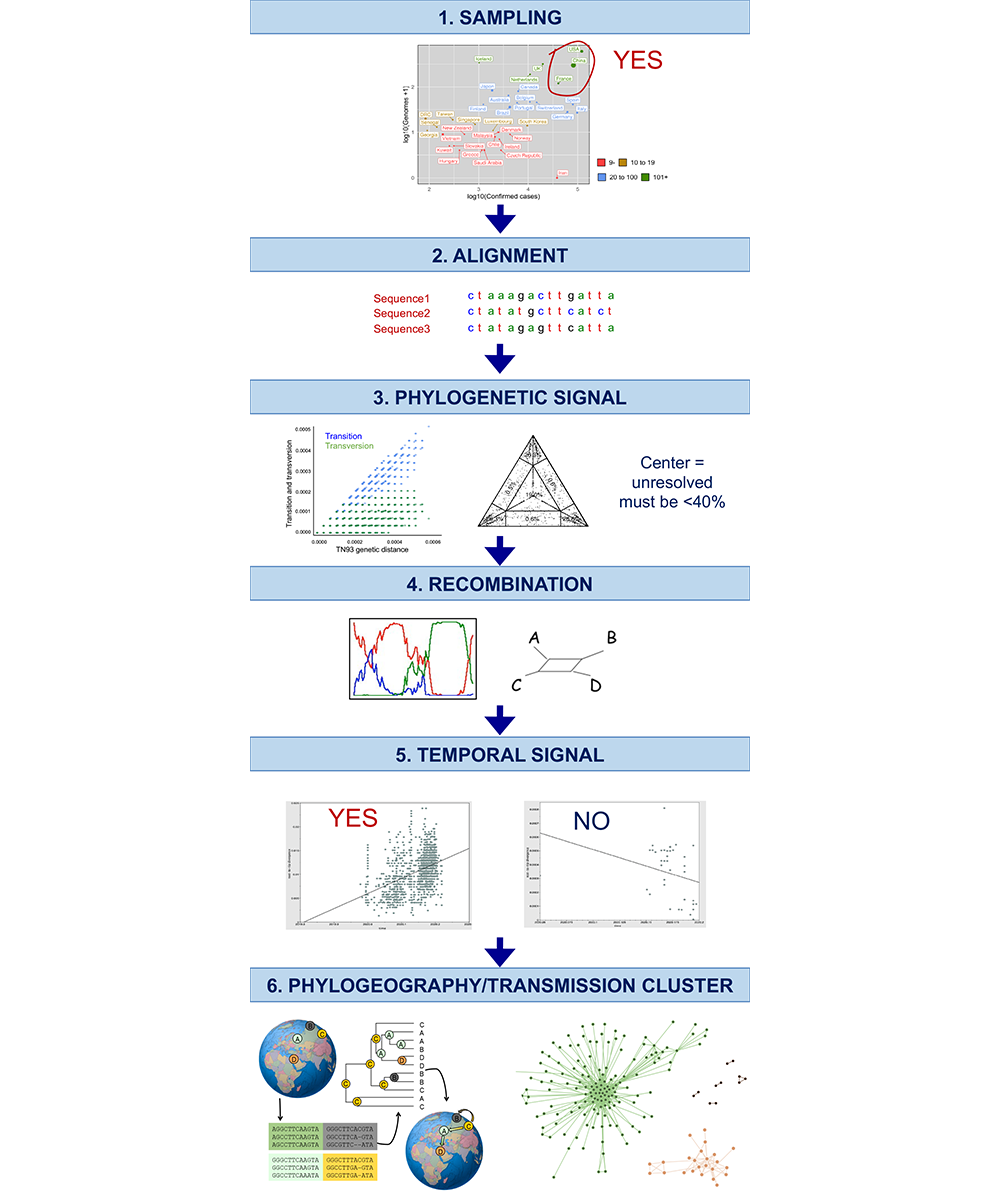

Supplement: Multimedia Appendix 3 [file publichealth_v6i2e19170_app3.doc]
